# Wessex Branch of the Association of Clinical Pathologists Abstracts of Meeting in Bristol, November 1984

**Published:** 1985-04

**Authors:** 


### Wessex Branch of the Association of Clinical Pathologists

Meeting held at Southmead Hospital, Bristol, 21st November 1984

The meeting was conducted under the Presidency of Dr. D. Betty Brownell, with Dr. K. A. V. Cartwright as Secretary. It was attended by pathologists from all disciplines in the South West Region. The following papers were presented during the Scientific Sessions.

### MUSCLE IN LYMPH NODES

**J. L. Channer and J. D. Davies, Royal Infirmary, Bristol**

There has been much debate as to whether muscle is present in normal lymph nodes and, if so, where. There are few records of muscle in abnormal lymph nodes. An incidental observation of muscular proliferation (Lott and Davies, this *Journal,* 1983, **98**, 90–91) in an axillary node draining breast carcinoma prompted this further investigation.

Examination by dissection microscopy and at higher magnification of inguinal and axillary lymph nodes for 'normal or reactive' (66 nodes) and breast-cancer axillary resections (344 nodes) showed that, overall, 8% of these superficial nodes displayed excessive smooth-muscle proliferation in the hilar region. These nodes were further classified as to the degree of smooth-muscle proliferation, and the quantity of blood vessels in their hila. A statistically significant relationship ($P = 0.01$) between these last two features was found. Additionally, a tendency was found for nodes (in cases where several were resected) to be involved simultaneously to the same degree. In the series the highest frequency of such changes was found in the inguinal nodes, which are well known to be especially susceptible to post-inflammatory fibrosis, from men.

In view of these findings, it is postulated that hilar smooth-muscle proliferation in superficial lymph nodes may be a marker of earlier inflammatory reactions, leading to regional lymphadenitis. It seems likely that the smooth muscle cells may be derived from the vessels in the hila of the lymph nodes. It is possible that smooth-muscle hyperplasia, at least in the circumstances studied, may reflect endogenous chemical stimuli released during inflammation.

### INFECTIONS OF INDWELLING CENTRAL VENOUS CATHETERS IN A PAEDIATRIC HAEMATOLOGY/ONCOLOGY UNIT

**N. C. Weightman, P. J. Darbyshire and D. C. E. Speller, Royal Infirmary and Children's Hospital, Bristol**

Forty-nine Broviac or Hickman indwelling central venous catheters were inserted in 36 children with various haematological diseases and malignancies. Sixteen patients suffered 29 episodes of catheter-related bacteraemia: 0.6 episodes per 100 days of catheter use. Infections occurred more commonly in inpatients with intensive use of the catheter than with relatively light outpatient use. Overall, 36.7% of catheters gave rise to one or more episodes of bacteraemia.

Coagulase-negative staphylococci accounted for 51% of the bacterial strains isolated, and *Staphylococcus aureus*, various species of *Streptococcus, Enterobacteriaceae, Acinetobacter* and *Candida glabrata* also occurred. Many of the infections (34%) were multiple, with up to six different strains being detected in single episodes, and discriminating examination of cultures is necessary.

Antibiotic administration via the catheter was successful in eradicating the infection in 72% of episodes, and was more likely to succeed in infections by single strains than in multiple infections. Four catheters were removed because of failure to overcome the infection.

Morbidity was often slight, particularly with coagulase-negative staphylococci, and no child died of infection with a central venous catheter *in situ*.

### SMALL CELL CARCINOMA OF LUNG: A SURVEY OF 193 RESECTED TUMOURS

**N. B. N. Ibrahim, J. C. Briggs, E. Andrianopoulos, K. Jayasingham and C. P. Forrester-Wood, Frenchay Hospital**

Accurate histological classification of pulmonary tumours is important in the assessment of the prognosis and choice of treatment. As both oat cell carcinoma and carcinoid tumours are believed to be neoplasms of a common cell ancestor, it is not surprising that occasionally there is an overlap between these tumours. Whilst carcinoid tumours behave in a relatively benign way or of low grade malignancy, oat cell carcinomas on the other hand have a distinct biological behaviour often associated with early invasion and metastasis.

Over the past two decades reports have indicated that occasionally carcinoid tumours with atypical histological features have been diagnosed as oat cell carcinomas. Furthermore, it has been suggested that examples of atypical carcinoid tumours may be encountered among long term survivors of what has been diagnosed as oat cell carcinomas. According to the latest W.H.O. classification of lung tumours (1981), small cell carcinoma of lung is subclassified into: oat cell carcinoma, intermediate cell type and combined oat cell carcinoma. There has recently been considerable controversy as to the significance and the validity of such a subclassification.

We reviewed 193 cases of small cell carcinoma of lung resected between 1969 and 1978 (with a minimum of 5 year follow-up). There was postoperative death in 26 cases; 8 cases were lost to follow-up. Of the remaining cases, 98 were oat cell carcinomas, 35 tumours were intermediate cell type, 8 tumours were combined oat cell carcinomas, 5 consisted of small and large cell carcinomas, and 13 tumours were reclassified as atypical carcinoids. The table summarises the follow-up results.

*Histological type and survival in resected small cell carcinomas of lung*

| | No. of cases | Mean survival (months) | >2 years survival No. of cases |
|---|---|---|---|
| Oat cell | 98 | 17.3 | 15 (15.3%) |
| Intermediate | 35 | 27.7 | 8 (22.9%) |
| 'Atypical carcinoid' | 13 | 62.5 | 6 (46.1%) |
| Combined oat | 8 | 5.7 | — |
| Small and large | 5 | 27.2 | 1 (20%) |

This table does not include postoperative deaths (26 cases) and 8 cases which were lost to follow-up.

Thirty patients lived more than 2 years and, of these, 22 lived more than 5 years. We conclude that subclassification of small cell anaplastic carcinoma is of prognostic significance. The findings of 13 atypical carcinoids in our series is similar to published experience of some other workers in this field.

### THE SECRETORY IMMUNE SYSTEM IN THE BREAST AND COLON: ITS RELEVANCE TO NEOPLASIA

**S. Z. Al-Sam and J. D. Davies, Royal Infirmary, Bristol**

The production and immuno-localisation of secretory component (Sc), normally present in the secretory IgA, the IgA and J chain were studied by the indirect immunoperoxidase technique performed on formalin-fixed paraffin-embedded material. The results demonstrate that the production of Sc and the active transport of IgA and J chain across the epithelial membrane is seen both in normal colonic mucosa and lactating breasts. Loss of production of Sc and impairment of transport IgA is seen in colonic adenomas and carcinoma. The degree of impairment of this immune system, in association with colonic neoplasms, is inversely proportional to the degree of dysplasia of the tumour cells. Similar findings are seen in all the breast carcinomas examined so far. However, the loss of function is absolute even in the well differentiated forms of breast carcinoma. In both systems the abnormality affecting this immune system is localised to the area of neoplasia, whereas the adjacent normal epithelium retains its full capacity to produce Sc and to transport IgA.

It is felt that this study may contribute to the detection of the site of neoplastic transformation in the breast lesions. Controversy in this field may be resolved by this immunohistochemical approach. However it seems probable that conventional morphology, allied to immunohistochemistry, will be necessary to relate the findings to earlier subjective judgements in breast pathology.

### THE BRISTOL MUMMY

**N. J. Brown, Southmead Hospital, Bristol**

In 1981 a deteriorating 3000-year-old Egyptian mummy from Bristol Museum was unwrapped and dissected by a multidisciplinary scientific, medical and dental team. Scientific studies of the wrappings, embalming materials and enmeshed insects and seeds were rewarding but pathological examination was disappointingly restricted by the decrepit state of much of the body and the absence of all viscera. Radiological evidence of asymmetrical spinal arthritis (Forrestier's disease) and the state of the teeth were consistent with collateral infirmation that the subject was a middle-aged man. Attempts to establish blood group and tissue types were unsuccessful. Cultures yielded no bacterial growth but Clostridial spores were demonstrated by electron microscopy; the embalming resin had antibiotic activity. Good finger-prints and scanning electron micrographs of skin and hair were obtained, showing evidence of recent shaving. Paraffin sections of skin and blood vessels after rehydration showed very well preserved elastic tissue and evidence of atherosclerosis. Good sections of bone and cartilage were also obtained. Death was probably due to natural causes rather than to trauma. Advanced putrefaction before mummification may have led the embalmers to discard the viscera, which would normally have been preserved in a package or canopic jar with the body.

### A COMPUTERISED MELANOMA REGISTRY

**J. C. Briggs and N. B. N. Ibrahim,**
**Frenchay Hospital, Bristol**

Since December 1980 the routine work of Frenchay's Histopathology Department has been performed with increasing use of computerisation. This has been centred on a word processing package, to which has been added many data processing programmes, all of which were developed by the Department of Medical Physics. One 'add on' transferred the Melanoma Registry from its filing card index to a specific computer data bank. The link between this data bank, which currently contains up to 49 fields of information about more than 1500 patients, allows a 'Mailshot' automatically to produce follow-up questionnaires to patients' GPs. Selectively, each year the GPs of all living patients are contacted. (Of all 1500 cases only 22 are classified 'lost'; even the Office of Population Census and Surveys has no knowledge of these people. Several are known to have gone abroad.)

This information bank has implications for clinical management and research. A study, conducted around an automatic mailing to patients known to be alive after a recurrence, revealed self-detection of the recurrence in about 85%. Additionally the study showed recurrences to occur overwhelmingly in the first five years after presentation. It seems, therefore, unrewarding to follow up patients in hospital clinics for longer than five years. This clinical practice is being instituted.

Melanoma prognosis is intimately related to the thickness of the lesion and to the depth of extension into the underlying skin. Both parameters have disadvantages and, in an attempt to overcome these, the thickness of uninvolved dermis beneath the tumour was measured. A ratio of this thickness to that of the tumour itself was produced but, unfortunately, gave no better prognostic guide than the tumour thickness itself.

The type of data bank described in this study is applicable to almost any disease.

### JUST HOW FALLIBLE ARE FROZEN-SECTION DIAGNOSES?

**E. K. Dankwa and J. D. Davies,**
**Royal Infirmary, Bristol**

Frozen-section diagnosis is occasionally used during surgical operations. Today, with ancillary preoperative diagnostic methods, it is less frequently employed than was the case 15 to 20 years ago. However such diagnosis remains important, since therapeutic decisions may be taken as a result of laboratory assessment.

In the past 10 years a record has been kept in the Bristol Royal Infirmary of all such frozen-section diagnoses. During this period over 60,000 surgical requests were received. Of these a consecutive series of 1000 frozen-section specimens from operating theatres were reviewed. Comparison was made between the 'hot' diagnosis issued, and that sent out on the basis of subsequent paraffin block examinations.

The overall accuracy of frozen-section diagnosis, according to the criteria described above, was 98.6%. Most cases received, and most errors occurred in breast lesions. The material was analysed according to the source of the error. These fell into those due to technical imperfections, those arising from the focal nature of the lesions, and those that followed pathological misinterpretation. Further, errors were divided into those that were unavoidable (most in this category were due to associated inflammation or the focal nature of the tumour), and those that were regarded as potentially avoidable. Important amongst this latter group were cases due to morphological misinterpretation. Happily no false-positive misdiagnosis of malignancy was made in the 10-year period. The rare cases involving morphological misinterpretation merit further consideration.

